# Quercetin Induces Mitochondrial Mediated Apoptosis and Protective Autophagy in Human Glioblastoma U373MG Cells

**DOI:** 10.1155/2013/596496

**Published:** 2013-11-28

**Authors:** Hyeonji Kim, Jeong Yong Moon, Kwang Seok Ahn, Somi Kim Cho

**Affiliations:** ^1^Faculty of Biotechnology, College of Applied Life Sciences, Jeju National University, 66 Jejudaehakno, Jeju 690-756, Republic of Korea; ^2^Subtropical Horticulture Research Institute, Jeju National University, Jeju 690-756, Republic of Korea; ^3^Department of Oriental Pathology, College of Oriental Medicine, Kyung Hee University, 1 Hoegi-Dong dongdaemun-gu, Seoul 130-701, Republic of Korea

## Abstract

Quercetin is a dietary flavonoid with known antitumor effects against several types of cancers by promoting apoptotic cell death and inducing cell cycle arrest. However, U373MG malignant glioma cells expressing mutant p53 are resistant to a 24 h quercetin treatment. In this study, the anticancer effect of quercetin was reevaluated in U373MG cells, and quercetin was found to be significantly effective in inhibiting proliferation of U373MG cells in a concentration-dependent manner after 48 and 72 h of incubation. Quercetin induced U373MG cell death through apoptosis, as evidenced by the increased number of cells in the sub-G1 phase, the appearance of fragmented nuclei, decreased mitochondrial membrane potential, proteolytic activation of caspase-3 and caspase-7, an increase in caspase-3 and 9 activities, and degradation of poly(ADP-ribose) polymerase protein. Furthermore, quercetin activated JNK and increased the expression of p53, which translocated to the mitochondria and simultaneously led to the release of cytochrome c from mitochondria to the cytosol. We also found that quercetin induced autophagy. Pretreatment with chloroquine, an autophagy inhibitor, strongly augmented apoptosis in U373MG cells, indicating that quercetin induced protective autopagy in U373MG cells.

## 1. Introduction 

Glioblastoma is the most common type of primary brain tumor in adults and the most lethal and least successfully treated tumor. The low absolute incidence combined with high morbidity, poor response rate, and short survival time poses practical problems for clinical trial execution [1]. Less than 30% of patients suffering from this devastating disease survive 12–15 months, even after receiving multimodal treatments such as surgical resection, combined chemotherapy and radiotherapy, and adjuvant chemotherapy [2]. These observations underscore the need for alternative therapies to prevent and effectively treat glioblastoma.

Quercetin is an antioxidative flavonoid ubiquitously distributed in plants. Its anticancer effects have been attributed to antioxidative activity, inhibition of enzymes activating carcinogens, modification of signal transduction pathways, and interactions with receptors and other proteins [3]. Quercetin is an anticancer agent in many cancer models [4–12]. Several studies have reported that quercetin increases the efficacy of glioblastoma treatment by suppressing the PI-3-kinase-Akt pathway [13], inducing apoptosis by reducing X-linked inhibitor of apoptosis protein (XIAP) [14], blocking signal transducer and activator of transcription 3 (STAT3) [15], arresting cells at the G2 checkpoint of the cell cycle, and decreasing the mitotic index in glioma cells [16]. Such effects of quercetin in glioblastoma cells seem to be dependent on cell type because combined application of tumor necrosis factor-related apoptosis-inducing ligand (TRAIL) and quercetin strongly reduces viability of U87MG, U251, A172, and LN229 glioma cells but fails to reduce the viability of U373MG cells [17]. The cause of U373MG cell resistance to quercetin-TRAIL-mediated apoptosis is not fully understood. Inactivating p53 is not important in quercetin-TRAIL-mediated apoptosis, as both sensitive U251 and the completely resistant U373MG cells have p53 mutations.

Apoptosis is programmed cell death mediated by caspases, which are cysteine proteases that cleave target proteins at aspartic acid. p53 is a transcription factor that induces the expression of proapoptotic genes [18, 19], and activating apoptosis is an important mechanism in p53-induced tumor suppression. Mitochondrial localization of mutant p53 and evidence linking p53 transcription-independent or mitochondria-targeted apoptosis has received considerable attention. A fraction of p53 translocates to mitochondria prior to changes in the mitochondrial membrane potential, cytochrome c release, and activation of caspases [20–22]. A previous study suggested that cellular cross-talk may occur between mitochondrial and nuclear p53. Heyne et al. [23] suggested that mutant p53 exists as a monomeric protein in mitochondria, and Tang et al. [24] demonstrated that mutant p53 translocates to mitochondria in UVB-irradiated murine skin carcinoma cells. Mahyar-Roemer et al. [25] suggested that mutant p53 is present in mitochondria independent of apoptotic signals. Mihara et al. [26] reported that p53 binds to Bcl-xL via its DNA-binding domain and that mutant p53 R273H cannot bind Bcl-xL and is therefore unable to activate the direct mitochondrial pathway of apoptosis despite being localized in or at the mitochondria. However, mutant p53 can also induce apoptosis through a transcription-independent pathway, in which wild-type p53 and p53 mutants are transiently located to the mitochondria with changes in the mitochondrial membrane potential [27]. Furthermore, a few such transcriptionally impaired p53 mutants (e.g., the structural mutant R175H and the DNA contact mutants R273H and C277F) bind Bak in vitro, and this is correlated with their ability to oligomerize Bak and induce cytochrome c release from isolated mitochondria [28]. We questioned whether mutant p53 R273H affects mitochondrial functions in human glioma blastoma cells.

Here, we addressed the question of whether autophagy is intimately linked with apoptosis induced by quercetin in U373MG cells. Autophagy is an evolutionarily conserved and genetically programmed process that degrades long-lived cellular proteins and organelles. The role of autophagy in cancer is quite complicated and controversial. Autophagy is assumed to be tumor suppressive during cancer development but to contribute to tumor cell survival during cancer progression [29]. Alternatively, autophagy prevents tumor cells from dying by inhibiting apoptosis during nutritional deprivation, and the cells undergo apoptosis when autophagy is prevented [30–32]. Regardless of whether they promote cell survival or cell death, the two processes engage in complex and poorly understood molecular cross-talk [33] and inducing apoptosis and inhibiting protective autophagy have become an effective means of cancer therapy. With the aim of exploring the effective anticancer activity of quercetin, we reevaluated the induction of cell death by quercetin at various concentrations and incubation times and examined the role of autophagy in quercetin-induced apoptosis in p53-mutant U373MG cells. Our results demonstrated for the first time that quercetin suppresses the proliferation of U373MG cells by modulating apoptotic and autophagic fluxes. Quercetin-induced cytotoxicity of U373MG cells was enhanced when autophagy was blocked before quercetin treatment. Thus, inhibiting autophagy must be considered a therapeutic approach to reduce U373MG proliferation and as a promising strategy to sensitize cells to quercetin treatment.

## 2. Materials and Methods

### 2.1. Reagents

Dulbecco's modified Eagle's medium (DMEM), trypsin-EDTA, fetal bovine serum (FBS), penicillin, streptomycin, Alexa Fluor 488 Goat anti-rabbit IgG(H+L), and Hoechst 33342 dye were purchased from Invitrogen Life Technologies, Inc. (Grand Island, NY, USA). Dimethyl sulfoxide (DMSO) and MTT were obtained from Amresco (Cleveland, OH, USA). Quercetin, chloroquine, propidium iodide (PI), acridine orange, and LC3II antibody were purchased from Sigma Chemical Co. (St. Louis, MO, USA). JNK, phospho-JNK, p53, Beclin-1, cytochrome c, cleaved poly(ADP-ribose) polymerase (PARP), and caspase-3, -7, and -9 antibodies were acquired from Cell Signaling Technology (Beverly, MA, USA). Polyclonal anti-caspase-8 was purchased from R&D Systems (Minneapolis, MN, USA). The BD Mitoscreen (JC-1) kit was acquired from BD Biosciences (Franklin Lakes, NJ, USA). A BCA protein assay kit was obtained from Pierce (Rockford, IL, USA), and polyvinylidene fluoride (PVDF) membranes were purchased from Millipore (Bedford, MA, USA). Fluorescence Mounting Medium was purchased from DAKO (Ely, United Kingdom). All other reagents used were of analytical grade.

### 2.2. Cell Culture

Human glioblastoma U373MG cells were kindly provided by Professor Tae-Hoo Yi at the Department of Biotechnology, Kyunghee University, Republic of Korea [34]. The U373MG cells were cultured in DMEM containing 10% (v/v) heat inactivated FBS, 100 units/mL penicillin, and 100 **μ**g/mL streptomycin. The cells were maintained in a humidified 5% CO_2_ incubator at 37°C.

### 2.3. Cell Viability

The effect of quercetin on U373MG cell viability was determined by an MTT-based assay [35]. Briefly, exponential-phase cells were collected and transferred to a microtiter plate (1 × 10^4^ cells/mL). The cells were incubated with various concentrations of quercetin and/or chloroquine for 24, 48, and 72 h. Then, 0.1 mg of MTT was added to each well and incubated for 4 h at 37°C. The medium was removed, and DMSO (150 *μ*L) was added to each well to dissolve the formazan crystals. The plates were read immediately at 570 nm on a Sunrise microplate reader (Tecan, Salzburg, Austria). The percentage of viable cells was calculated based on the following formula: mean value of (control group − treated group/control group) × 100%. All results were assessed in triplicate at each concentration.

### 2.4. Cellular Morphology, Nuclear Fragmentation, and Acidic Vesicular Organelles

U373MG cells were placed in 12-well plates at 3 × 10^4^ cells/mL and treated with different concentrations of quercetin and/or pretreated with chloroquine. After 48 h, 10 **μ**M Hoechst 33342, a DNA-specific fluorescent dye or acridine orange, a lysotropic dye, was added to each well, and the plates were incubated for 10 min at 37°C. The stained cells were observed under an Olympus fluorescence microscope (Tokyo, Japan).

### 2.5. Flow Cytometric Analysis

Cells (1 × 10^4^ cells/mL) were plated in 60 mm plates and treated with quercetin (0–100 **μ**M) for 48 h. Then, the cells were harvested, washed with phosphate-buffered saline (PBS), fixed in 70% ethanol, rehydrated in 2 mM EDTA-PBS, treated with RNase A (25 ng/mL), and stained with propidium iodide (40 **μ**g/mL) for flow cytometry. The cells were stained with 10 **μ**M acridine orange, harvested, and maintained in 2 mM EDTA-PBS containing 10% FBS to detect autophagy. We followed the manufacturer's protocol for JC-1 mitochondrial membrane detection. In brief, treated cells were trypsinized and washed with 1× assay buffer, stained with JC-1 for 15 min at 37°C in a CO_2_ incubator, and washed twice with 1× assay buffer at room temperature. All analyses were performed using a FACScaliber flow cytometer (BD Biosciences). Data from 10,000 cells per sample were analyzed with CellQuest software (BD Biosciences). Each experiment was repeated at least three times.

### 2.6. Caspase Activity

Caspase-3 and -9 activities were measured using a colorimetric assay following the protocol of the commercially available kit from Sigma Chemical Co. and Biovision (San Jose, CA, USA), respectively. Briefly, cells were lysed after a 48 h quercetin treatment with or without chloroquine, and aliquots (10 **μ**L) of the supernatant were placed in a 96-well microplate containing reaction buffer. Substrate was added, and the microplate was incubated at 37°C overnight. Activity was monitored as the linear release of *p*-nitroaniline from the substrate and compared with a linear standard curve generated on the same microplate.

### 2.7. Cellular Fraction and Immunoblot Analyses

U373MG cells were collected and washed twice with cold PBS after treatment with various quercetin concentrations. The cells were then lysed in lysis buffer (50 mM Tris-HCl, pH 7.5, 150 mM NaCl, 1% Nonidet P-40, 2 mM EDTA, 1 mM EGTA, 1 mM NaVO_3_, 10 mM NaF, 1 mM DTT, 1 mM PMSF, 25 *μ*g/mL aprotinin, and 25 *μ*g/mL leupeptin) and kept in ice for 30 min. The lysates were centrifuged for 30 min at 13,000 rpm and 4°C, and the supernatants were stored at –70°C until use. Cytosolic and mitochondrial extracts were prepared using fraction lysis buffer (75 mM NaCl, 8 mM Na_2_HPO_4_, 1 mM Na_2_H_2_PO_4_, 250 mM sucrose, 1 mM EDTA, and 350 *μ*g/mL digitonin). Lysed cells were kept in ice for 10 min and then centrifuged for 15 min at 15,000 rpm and 4°C. The supernatant was the cytosolic fraction. After the pellet was washed with lysis buffer, it was lysed in lysis buffer to prepare whole lysates. Protein concentration was measured using a BCA Protein Assay kit. Aliquots of the lysates (30–70 **μ**g protein) were separated via 10–15% sodium dodecyl sulfate-polyacrylamide gel electrophoresis and transferred onto a PVDF membrane using glycine transfer buffer (192 mM glycine, 25 mM Tris-HCl, pH 8.8, and 20% methanol, v/v). After blocking with 5% nonfat dried milk, the membranes were incubated for 4 h with primary antibodies, followed by an additional 30 min incubation with secondary antibodies in milk containing Tris-buffered saline and 0.1% Tween 20. Human anti-caspase-3, -caspase-7, -caspase-8, cleaved PARP, cytochrome c, JNK, phospho-JNK, p53, HSP60, LC3II, and Beclin-1 antibodies were used at a 1 : 1,000 dilution as the primary antibodies, and horseradish peroxidase-conjugated goat antihuman IgG was utilized as the secondary antibody at a 1 : 5,000 dilution. The membranes were then exposed to X-ray film. Protein bands were detected using the WEST-ZOL plus Western blot detection system (Intron, Gyeonggi-do, Republic of Korea).

### 2.8. Statistical Analysis

All results are expressed as means ± standard deviations. A one-way analysis of variance was conducted using the SPSS ver. 12.0 for Windows, 2004 package (SPSS Inc., Chicago, IL, USA). *P* < 0.05 was considered significant. All assays were performed in triplicate.

## 3. Results and Discussion

### 3.1. Quercetin Induces Apoptotic U373MG Cell Death

**Figure 1:**
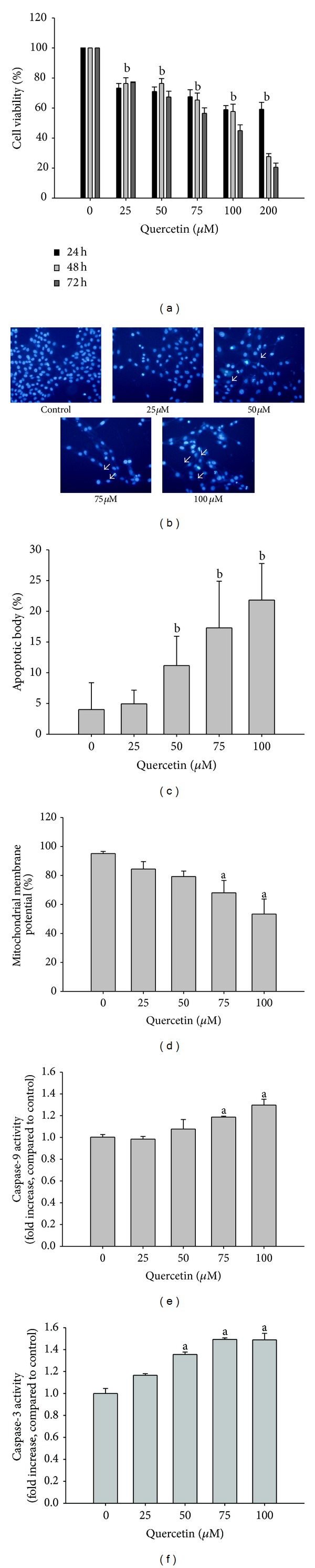
Quercetin inhibits cell growth and induces apoptosis. (a) Cell viability was determined by MTT reduction. U373MG human glioblastoma cells were treated with increasing doses of quercetin for different lengths of time (24–72 h). Data are the mean ± standard error for one experiment performed in triplicate. Values are the mean ± standard deviation (SD) of three independent experiments. ^b^ *P* < 0.01 compared to the control. (b) Treated cells were stained with nuclear Hoechst 33342 and visualized under a fluorescence microscope after a 48 h treatment. White arrows indicate apoptotic bodies. Representative areas were photographed with 200X magnification. (c) Graph for quantification of number of apoptotic bodies. Data are the mean ± standard error for one experiment performed in triplicate. ^b^ *P* < 0.01 compared to the control. (d) Flow cytometry analysis of JC-1 staining. Cells were trypsinized, stained with JC-1, washed, and analyzed by flow cytometry. The decrease in mitochondrial membrane potential percentage indicates that cells were undergoing mitochondrial dysfunction. (e) Caspase-9 and (f) caspase-3 activities in U373MG cells. Caspase activities were measured by a colorimetric assay and calculated as fold changes compared to the same control. The cells were treated with quercetin for 48 h ((b)–(f)). All data are the mean ± SD of three independent experiments. a: significantly different from the control, *P* < 0.05.

The MTT assay was performed to investigate the cytotoxic effects of quercetin on human glioblastoma U373MG cells. As shown in Figure 1(a), a 48 or 72 h incubation with quercetin reduced U373MG cell viability in a dose-dependent manner. A significant decrease in cell viability was observed after 48 (27.63%) and 72 h (20.52%) of incubation at a concentration of 200 **μ**M, whereas cell viability after a 24 h incubation was 59.17% (Figure 1(b)). Various morphological changes such as cell shrinkage and condensation as well as chromatin fragmentation were clearly noted during apoptotic cell death. U373MG cells treated with various concentrations of quercetin were examined by fluorescence microscopy after Hoechst 33342 staining to evaluate the effect of quercetin on the induction of apoptosis. As depicted in Figure 1(b), cells showed marked morphological changes such as condensed and fragmented chromatin and the formation of apoptotic bodies after treatment with 25, 50, 75, and 100 **μ**M quercetin. Furthermore, the induction of apoptosis was indicated by the accumulation of sub-G1-phase U373MG cells after quercetin treatment. A significant increase in sub-G1-phase cells was not observed in the 24 h treatment (from 1.02% at 0 **μ**M to 2.05% at 100 **μ**M). However, quercetin significantly increased the sub-G1 population concentration dependently after the 48 h treatment (from 0.46% at 0 **μ**M to 8.48% at 100 **μ**M; Table 1). The mitochondrial membrane potential was disrupted as a result of apoptosis (Figure 1(c)) in dose-dependent manner. Because caspases-9 and -3 are key executioners of apoptosis, we quantified their enzymatic activities using a commercially available kit. As a result, quercetin dose-dependently increased caspase-9 and -3 activities (Figures 1(d) and 1(e)). These results indicate that quercetin induces apoptosis in U373MG cells via the intrinsic pathway by activating caspase-3 and caspase-9.

### 3.2. Effects of Quercetin on Apoptosis-Related Protein Expression

**Figure 2:**
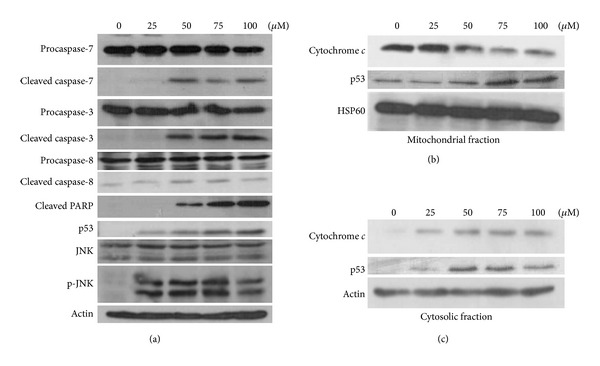
Expression levels of apoptosis-related proteins were analyzed by immunoblotting. (a) Cellular proteins, (b) mitochondrial fraction, and (c) cytosolic fraction were separated by sodium dodecyl sulfate-polyacrylamide gel electrophoresis and transferred to PVDF membranes. The membranes were probed with the indicated primary antibodies and then with horseradish peroxidase conjugated goat anti-rabbit IgG. Heat shock protein (HSP)60 was used as the loading control for the mitochondrial proteins (b). Actin was used as the internal control (a) and loading control for the cytosolic proteins (c).

The expression of apoptosis-related proteins and activation of caspases following quercetin treatment were examined by Western blotting to further determine the mechanism of quercetin-induced apoptosis. The protein level of the caspase-7 and -3 precursor decreased, and proteolytically cleaved caspase-7 and -3 as well as PARP levels increased, although not much change was observed in the caspase-8 level (Figure 2(a)). JNK was activated, and p53 protein and phosphorylation levels dose-dependently increased following quercetin treatment (Figure 2(a)). JNK is one of the mitogen-activated protein kinases (MAPKs) that regulate cell proliferation, differentiation, and apoptosis [36]. JNK directly or indirectly modulates the expression levels of p53 and its target genes [37]. Evidence suggests that p53 induces cell death by several molecular pathways involving the activation of target genes and transcriptionally independent direct signaling [21]. In our study, p53 expression increased concentration dependently, but the level of the p53 downstream protein, PUMA, remained unchanged (data not shown) because U373MG is a p53 mutant cell line. Our result suggests that mutant p53 in U373MG cells can induce apoptosis through a transcription-independent pathway, as the level of cytochrome c increased in the cytosolic fraction, whereas the level of cytochrome c decreased in the mitochondrial fraction (Figures 2(b) and 2(c)) with a decrease in mitochondrial membrane potential (Figure 1(c)). Although additional experiments are required to determine whether the p53-Bcl-2/xL interaction occurs to fully understand quercetin-induced death of the p53 mutant glioblastoma-derived cell U373MG cells, we speculate that our results are similar to previous data in C33A cells in which the p53 mutant was transiently located to the mitochondria with changes in the mitochondrial membrane potential [27].

### 3.3. Quercetin Induces Autophagy in U373MG Cells. 

**Figure 3:**
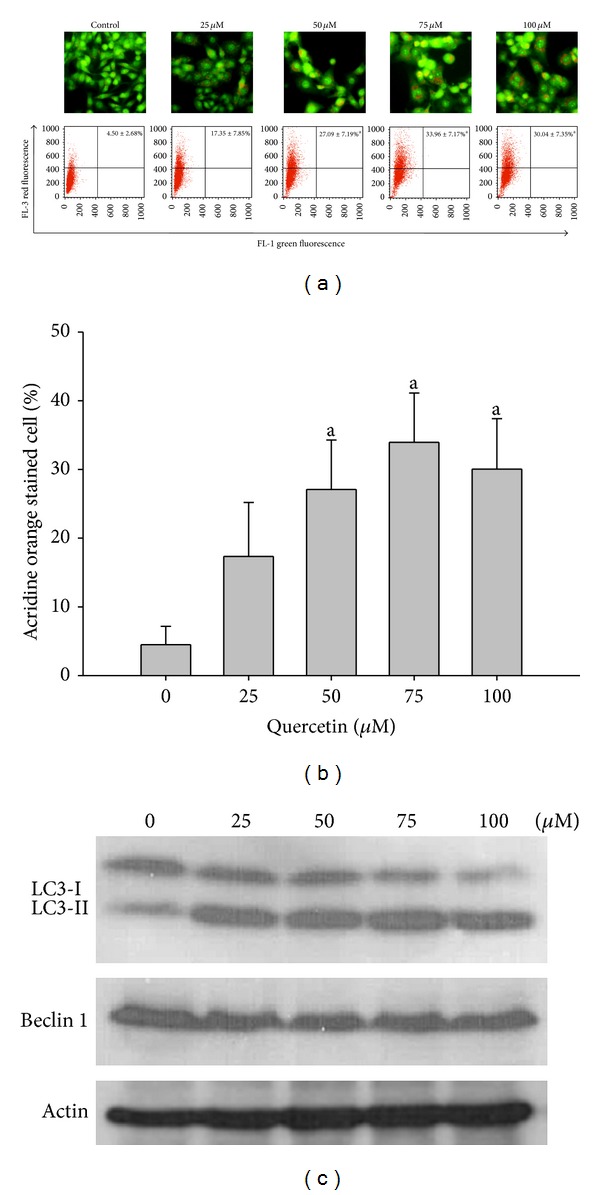
Quercetin induces autophagy in U373MG cells. The cells were treated with various concentrations of quercetin for 48 h. (a) Analysis of the formation of autophagosomes and autolysosomes by fluorescence microscopy and flow cytometry. Representative areas were photographed with 200X magnification. (b) Quantification of acridine orange-positive cells. (c) Western blotting using antibodies specific for LC3, Beclin-1, and actin. Results shown are representative of three independent experiments. Images were captured using a fluorescence microscope. Results shown are representative of at least three replicates. All data are the mean ± standard deviation of three independent experiments. a, significantly different from the control, *P* < 0.05.

We examined the effects of quercetin on other cellular responses associated with cell death to better understand its anticancer effect. Acridine orange staining was used to analyze the formation of acidic vesicular organelles (AVOs) or autophagolysosome vacuoles, which occur as a result of fusion between autophagosomes and lysosomes as a key feature of autophagy [38]. Large numbers of AVOs were detected in U373MG cells treated with quercetin (Figure 3(a)). The FACS analysis showed that AVOs formed in 17.35% of U373MG cells treated with 25 **μ**M quercetin and in 33.9% of U373MG cells treated with 75 **μ**M quercetin and that a slight decrease (30.04%) occurred in U373MG cells treated with 100 **μ**M quercetin (Figure 3(b)). These data are consistent with the results of our Western blot analysis of the autophagy marker protein LC3. Conversion of the lipidated form of LC3 (LC3-I) to LC3-II, which is an autophagosomal marker, is due to localization and aggregation of LC3-II in autophagosomes [39]. Quercetin induced the processing of full-length LC3-I (18 kDa) to LC3-II (16 kDa) dose-dependently at concentrations up to 75 **μ**M, but a slight decrease was observed in 100 **μ**M treated cells (Figure 3(c)). These results indicate that autophagy is less predominant in U373MG cells treated with a high concentration of quercetin. In contrast to LC3, Beclin-1 expression remained unchanged (Figure 3(c)). Although Beclin-1 plays an important role in autophagy, several studies have revealed that autophagy can occur in a Beclin-1-independent manner [40, 41]. We analyzed the cells stained with fluorescent LC3 antibodies to confirm that quercetin induced autophagy. Quercetin reportedly can induce autophagy in many different cell lines such as gastric, bladder, and colon cancer cell lines [6, 42, 43]. However, a recent study reported that quercetin significantly induces apoptosis but has no effect on inducing autophagy in the T98G glioma cell line [44], highlighting the heterogeneity of glioma cell biology; therefore, comprehensive multicell line studies are needed. P53 typically regulates autophagy via transcription-dependent and -independent mechanisms. A spectrum of p53 target genes such as DRAM and AMPK positively regulates autophagy in a transcription-dependent manner [45]. However, several studies have reported that cytoplasmic p53 inhibits autophagy in a transcription-independent manner through a poorly investigated mechanism [46–48]. Further investigation is required to determine whether increase in the cytosolic mutant p53 following quercetin treatment in U373MG cells represses autophagy and promotes apoptosis through translocation to mitochondria.

### 3.4. Inhibition of Autophagy by Chloroquine Promotes U373MG Cell Apoptosis

**Figure 4:**
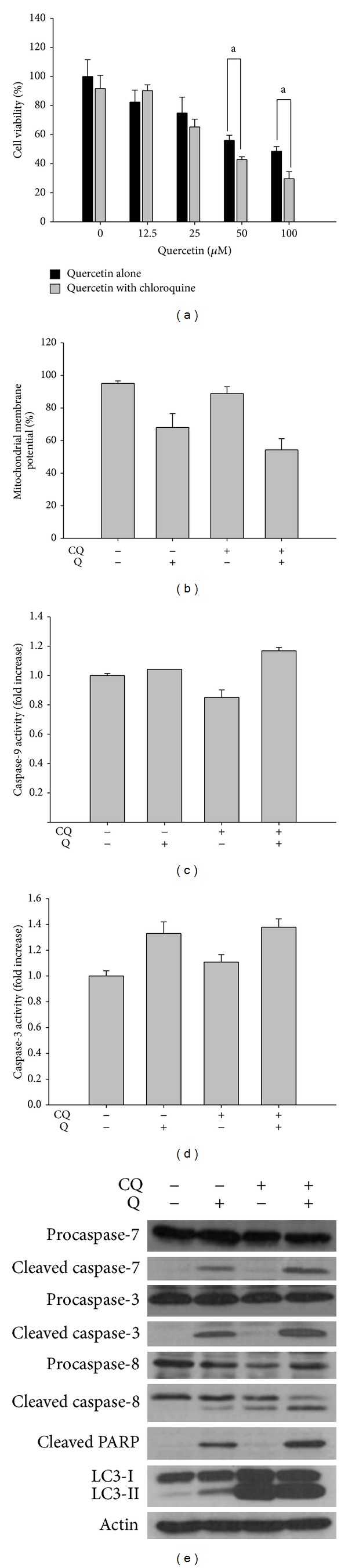
Inhibition of autophagy by chloroquine enhances apoptotic cell death. Cells were treated with 50 **μ**M chloroquine for 2 h before quercetin treatment. (a) Cell viability was measured by the MTT assay. Values are the mean ± standard deviation (SD) of three independent experiments. a: significantly different from the control, *P* < 0.05; b: significantly different from the control, *P* < 0.01. (b) The percentage of cells undergoing mitochondrial dysfunction. Treated cells were trypsinized, stained with JC-1, washed, and analyzed by flow cytometry. (c) Caspase-9 and (d) caspase-3 activities. All data are the mean ± SD of three independent experiments. a: significantly different from the control, *P* < 0.05. (e) Analysis of apoptosis-related protein and LC3II expression by Western blotting. CQ: 50 **μ**M chloroquine; Q: 75 **μ**M quercetin.

Several studies have shown that autophagy and apoptosis interact. Some proteins, already known as autophagy proteins, have dual functions in autophagy and apoptosis [4, 35]. To further analyze whether the autophagy signal induced by quercetin is prosurvival or prodeath, we treated U373MG cells with chloroquine, an inhibitor of autophagy, for 2 h before quercetin treatment. Chloroquine induces apoptosis in glioma cell lines via the p53 pathway [49]. The chloroquine treatment alone showed no effect on cell viability but a combined treatment with quercetin decreased cell viability compared to that of quercetin treatment alone (Figure 4(a)). Furthermore, pretreatment with chloroquine plus quercetin resulted in significant sub-G1 phase cell cycle arrest; the percentage of sub-G1 cells was 1.45%, 5.18%, and 18.68%, in U373MG cells treated with quercetin alone, chloroquine alone, and pretreated with chloroquine plus quercetin, respectively (Table 2). The mitochondrial membrane potential values were 68.0% and 54.3% following treatment with 75 **μ**M quercetin alone and after pretreatment with chloroquine plus quercetin, respectively (Figure 4(b)). Caspase-9 and -3 activities increased slightly in the combined treatment compared to those of quercetin treatment alone (Figures 4(c) and 4(d)). Western blot results showed a similar tendency in the cell cycle distribution. We also confirmed that inhibiting autophagy promoted apoptosis by increasing cleavage of caspases-3 and -7 and PARP (Figure 4(e)). In particular, the proteolytic cleavage of procaspase-8, which was not detected following quercetin treatment alone, rose after chloroquine pretreatment. Taken together, these data strongly suggest that inhibiting autophagy enhances apoptotic cell death induced by quercetin in U373MG cells.

## 4. Conclusion 

We demonstrated that quercetin induced cell death in the human glioblastoma U373MG cell line through an apoptotic pathway, which was confirmed by cleavage of caspases, PARP, and sub-G1 phase cell cycle arrest. Quercetin also activated JNK and modulated p53 expression accompanied by increased translocation of p53 to the mitochondria. The induction of autophagy in U373MG glioblastoma cells by quercetin was confirmed through acridine orange staining and conversion of LC3II. Furthermore, pretreatment with chloroquine enhanced intrinsic and extrinsic apoptotic cell death induced by quercetin, indicating that quercetin induced protective autophagy in U373MG cells. These results suggest that quercetin, in a combined treatment with an autophagy inhibitor, may be an excellent therapeutic approach to reduce U373MG proliferation and could be a promising strategy to sensitize cells to quercetin treatment.

## Figures and Tables

**Table 1:** The percentage of U373MG cells in the sub-G1 fraction after treatment with different doses of quercetin for 24 and 48 h.

Concentration (*μ*M)	24 h	48 h
	Sub-G1 phase (%)	Sub-G1 phase (%)
Control	1.02 ± 0.35	0.5 ± 0.31
25	1.78 ± 0.89	1.71 ± 0.53
50	2.33 ± 1.17	2.26 ± 0.86
75	1.65 ± 0.65	6.66 ± 0.79^a^
100	2.05 ± 0.93	8.48 ± 0.40^a^

All data are the mean ± SD of three independent experiments. ^a^Significantly different from the control, *P* < 0.05.

**Table 2:** The percentage of U373MG cells in the sub-G1 fraction after treatment with 75 *μ*M quercetin alone for 48 h, 50 *μ*M chloroquine alone for 2 h, or quercetin for 48 h plus pretreatment with chloroquine for 2 h.

Treatment	Sub-G1 phase (%)
Control	2.21 ± 1.1
Quercetin alone	1.45 ± 0.21
Chloroquine alone	5.18 ± 0.96
Chloroquine + quercetin	18.68 ± 3.02
